# Asymmetries in Gender-Related Familiarity with Different Semantic Categories. Data from Normal Adults

**DOI:** 10.3233/BEN-120277

**Published:** 2013

**Authors:** Guido Gainotti, Pietro Spinelli, Eugenia Scaricamazza, Camillo Marra

**Affiliations:** ^1^Center for Neuropsychological ResearchDepartment of Neurosciences of the Policlinico Gemelli/Catholic University of RomeRomeItaly; ^2^IRCCS Fondazione Santa LuciaDepartment of Clinical and Behavioral NeurologyRomeItaly

**Keywords:** Semantic categories, gender-related asymmetries, living beings, familiarity ratings, social roles

## Abstract

The mechanisms subsuming the brain organization of categories and the corresponding gender related asymmetries are controversial. Some authors believe that the brain organization of categories is innate, whereas other authors maintain that it is shaped by experience. According to these interpretations, gender-related asymmetries should respectively be inborn or result from the influence of social roles. In a previous study, assessing the familiarity of young students with different 'biological' and 'artefact' categories, we had observed no gender-related difference on any of these categories. Since these data could be due to the fact that our students belonged to a generation in which the traditional social roles have almost completely disappeared, we predicted that gender-related asymmetries should be found in older men and women. The familiarity of young and elderly men and women with various semantic categories was, therefore, studied presenting in the verbal and pictorial modality different kinds of living and artefact categories. Results confirmed the hypothesis, because elderly women showed a greater familiarity for flowers and elderly men for animals. These findings are consistent with the hypothesis assuming that gender-related asymmetries for different semantic categories is due to the influence of gender-related social roles.

## 1. Introduction

In a recent, authoritative review of current models of conceptual representations in long-term memory, Kiefer and Pulvermuller [21] have claimed that one of the main dimensions along which semantic categories can be processed concerns the 'innate vs experience-dependent' distinction.

The main proponents of the notion of an 'innate' categorical organization of conceptual knowledge are Caramazza and coworkers [10–12], who have proposed that category-specific impairments for animals, plant life and artefacts reflect an innate categorical organization of neural networks, shaped by natural selection to support rapid identification of objects very relevant for survival. At variance with this model, several authors (e.g. [20,34]) have shown that the acquisition of conceptual representations for novel objects is experience-dependent and that concepts are represented in sensory and motor networks through the learning-based formation of cortical cell assemblies.

An area of the neuropsychological research where the debate between 'innate' and 'experience-dependent' models is particularly dynamic is that of gender-related asymmetries in the ability to process different semantic categories (e.g. animals vs plant-life vs artefacts). These asymmetries are documented by a sizable body of data, obtained both in normal subjects and in brain-damaged patients. For instance, significant gender-related asymmetries about these categories have been observed in normal subjects gathering normative naming data from adults and children [15,32], evaluating familiarity ratings for various categories [1] and studying the age of acquisition of common names [3, 15]. Similar asymmetries have been found on speed-

ISSN 0953-4180/13/$27.50 © 2013 – IOS Press and the authors. All rights reserved ed naming [26] and identification [27] tasks, on name generation tasks [29], on semantic fluency tasks [7,8, 31], on object decision tasks [4] and using the semantic priming paradigm [5]. Asymmetries consistent with those observed in normal subjects have also been found in brain-damaged patients [9,16,17,23,25,30,31,33]. In these studies a relative sparing of categories showing a gender-related advantage in normal subjects has usually been observed. The precise pattern of the categories more easily processed by men and women and the nature of these gender-related asymmetries are, however, still controversial. As for the categories more easily processed by men and women, most authors (e.g. [8, 23,25–30]) report that males are more proficient with artefacts and females with biological stimuli. Other authors have, however, argued that the pattern of gender-related asymmetries is more complex, because, within the living categories, men seem more proficient with 'animals' and women with fruit and vegetables [1,32, 29,16,17,3,7,33,35]. Furthermore, within the artefact categories men seem to fare better with tools and women with 'furniture' and 'kitchen utensils' [1,31,33].

The models offered to account for gender-related differences have generally focused on asymmetries observed between artefact and biological categories and (within the latter) between animals and plant-life categories and have rarely considered those concerning artefacts.

The first interpretation of these asymmetries is based on the innate model of the 'domains of knowledge' [9–12] which assumes that natural selection produced specialized and dissociable neural networks for animals and plant life, because these two biological categories play a different but equally important role in human survival. An interpretation of sex-related asymmetries coherent with this general hypothesis was offered by Laws [27,29] and developed by Laiacona et al. [24]. According to Laws [27,29], a greater development of brain circuits dealing with tools and animals in men and with fruits and vegetables in women could be produced by the main subsistence activities of men (hunting) and women (gathering). Refining this line of thought, Laiacona et al. [24], proposed that the evolutionary pressures which prompted the development of different brain networks dedicated to animals and plant life might also have provided each gender with more efficient cognitive representations of their main foraging targets (i.e. animals for men and fruits and vegetables for women). A different, experience-dependent interpretation of gender-related asymmetries was proposed by Gainotti [16,17] on the basis of a reviews of the influence that gender and lesion location could have on naming disorders for animals, plant life and artefacts. This author suggested that only the distinction between living and non living things reflects an inborn anatomically-based categorical organization, whereas the discrepancy (within living entities) between animals and plant life might be due to social roles related familiarity factors, namely to men's greater familiarity with animals and women's with fruits and vegetables. The hypothesis that gender-related asymmetries may be experience-dependent is supported by the dissociations observed within the artefact categories, in which men are more proficient with tools and women with 'furniture' and 'kitchen utensils' [1,31,33]. The same hypothesis is, however, seemingly at variance with results recently obtained in young men and women by Moreno-Martinez et al. [33], studying semantic fluency on different subcategories of living and non-living things and by Gainotti et al. [18] assessing their familiarity with different 'biological' and 'artefact' categories. No difference was, indeed, observed in these studies between young males and females on any of the categories taken into account. These unexpected results could, perhaps, be due to the fact that young men and women included in the studies of Moreno-Martinez et al. [33] and of Gainotti et al. [18] were undergraduate students, belonging to a generation in which the traditional social roles have almost completely disappeared. An argument in favour of this point comes from the fact that in the above mentioned study of Moreno-Martinez et al. [33], differences consistent with the importance of social-role related familiarity factors have been observed in an elderly group. Older females showed, indeed, greater fluency for flowers, vegetables and kitchen utensils, whereas elderly males showed better fluency for musical instruments. In the present investigation we, therefore, used a procedure very similar to the one adopted in our previous study, to evaluate the familiarity of elderly men and women with subcategories of living and the non-living things. Our prediction was that we should find at the level of familiarity the same results obtained by Moreno-Martinez et al. [33], on a semantic category fluency task, namely significant (and consistent with the experience-dependent hypothesis) gender-related asymmetries in our elderly population, contrasting with the absence of gender-related asymmetries previously observed in the young group. In particular, on the basis of social-role related familiarity factors and of results obtained in elderly subjects by Moreno-Martinez et al. [33], and by Albanese et al. [1], studying familiarity ratings for various categories in an adult population (mean age 48.3 yrs), we advanced the following predictions: (a) within the living categories, men would show a greater familiarity for animals and women for flowers, fruits and vegetables; (b) within the non-living categories, men would show a greater familiarity for tools and women for furniture.

A second issue that we intended to check in our study was the possible interaction between familiarity assessment and presentation of stimuli in the verbal or in the pictorial modality, because, according to some authors (e.g. [2,6,22,36]) women are more verbal than men and tend to rely more on verbal than on perceptual data, whereas a bias between verbal and perceptual modalities is usually not reported in men. In our study stimuli were, therefore, presented both in the verbal (written words) and in the pictorial modality and familiarity ratings were analysed both lumping together and considering separately responses given after presentation of verbal and pictorial stimuli.

## 2. Experimental procedure

### *2.1. Materials*

Data were collected using 2 booklets, containing each 49 sheets with a standardized format, but with a different item heading each sheet. Each item was represented in one booklet by the picture of the target stimulus and in the other booklet by the corresponding written word printed in big capital letters. This presentation of stimuli both in the verbal and in the pictorial modality was made to control the influence that the verbal or pictorial nature of the stimuli could have on the subjective rating of familiarity The items consisted of 12 animals (6 domestic and 6 wild animals), 16 plant life items (6 flowers, 5 vegetables and 5 fruits) and 21 artefacts (5 pieces of furniture, 5 vehicles, 5 clothing and 6 tools). These items were the same used in our previous investigations [18,19]. For each stimulus, subjects were requested to evaluate the familiarity that they had with that object, using a Likert scale, whose scores ranged from 0 and 7, where 0 denoted 'no familiarity' and 7 a 'very high familiarity'. No subject had difficulty understanding the task, evaluating how familiar each stimulus was.

### *2.2. Subjects*

The study was conducted on 98 normal elderly people (49 males and 49 females) of age ranging between 60 and 84 years and with an educational level ranging between 5 and 13 years of schooling. One half of the males and one half of the females rated their familiarity with stimuli presented in the written modality, whereas the other half evaluated their familiarity with stimuli presented in the pictorial modality. Subjects were recruited among the caregivers of patients afferent at the Neuropsychology Centre of the Policlinico Gemelli or in structures for independent old people of the city of Rome who fulfilled the following criteria: (a) absence of neurological, medical or psychiatric disorders that could influence their cognitive functions; (b) absence of severe visual and auditory disorders; (c) age and education corrected MMSE scores within normal range. No significant differences were found among the 4 groups of elderly people enrolled in our study, with respect to their main demographic variables of age and educational level and their corrected MMSE scores. Furthermore, no interactions were observed between age, years of schooling and corrected MMSE scores. Data obtained were compared with a previous gathered population study of 98 normal young people (49 males and 49 females) of age ranging between 20 and 30 years and with an educational level ranging between 12 and 15 years of schooling. This young population study was already taken into account in our previous investigation [18]. Data included in this manuscript were obtained after approval of the Ethical Committee of the Policlinico Gemelli and written informed consent was obtained from all participants. No financial or other relationships that could be interpreted as a conflict of interest affects this paper.

## 3. Results

### *3.1. Familiarity of the various categories for young and older males and females irrespectively of the presentation modality*

Our first analysis consisted in evaluating the familiarity ratings of young and older males and females for the various categories of artefact and biological stimuli, irrespectively of the verbal or pictorial presentation modality. Results of this analysis are reported in Table 1.

Data were treated by means of a Three Way ANOVA considering as independent variables gender, age and the different semantic categories and as dependent variable the familiarity scores. A significant effect was found for the factor 'age' ($F_{(1-160)}$: 48.53; $p <$

Table 1
Familiarity of young and elderly men and women for the various categories of knowledge

| | Women | | Men | | p-level | |
|---|---|---|---|---|---|---|
| | Young | Elderly | Young | Elderly | Gender-related comparisons in young people | Gender-related comparisons in elderly people |
| Fruits | *6.45 (0.15)* | 6.32 (0.35) | *6.25 (0.24)* | 6.22 (0.29) | *0.75* | *0.51* |
| Vegetables | *6.25 (0.24)* | 6.01 (0.28) | *6.09 (0.28)* | 5.83 (0.33) | *0.67* | *0.24* |
| Flowers | *5.53 (0.62)* | 5.62 (0.62) | *5.30 (0.66)* | 5.07 (0.38) | *0.61* | *0.038* |
| Pets | *5.65 (0.42)* | 4.94 (0.76) | *5.58 (0.38)* | 5.29 (0.51) | *0.89* | *0.17* |
| Wild animals | *5.21 (0.29)* | 3.24 (0.77) | *4.76 (0.27)* | 3.72 (0.40) | *0.12* | *0.05* |
| *Living* | *5.82 (0.58)* | 5.16 (1.2) | *5.59 (0.68)* | *5.17 (0.94)* | *0.17* | *0.96* |
| Furniture | *6.54 (0.16)* | 6.23 (0.66) | *6.37 (0.22)* | 6.10 (0.53) | *0.75* | *0.61* |
| Clothes | *6.44 (0.22)* | 5.79 (0.79) | *6.24 (0.22)* | 5.79 (0.69) | *0.71* | *0.99* |
| Vehicles | *5.89 (0.52)* | 4.70 (1.04) | *5.71 (0.72)* | 5.13 (1.01) | *0.89* | *0.36* |
| Tools | *6.33 (0.35)* | 6.06 (0.78) | *6.23 (0.40)* | 6.26 (0.37) | *0.86* | *0.45* |
| *Non-living* | *6.30 (0.40)* | 5.71 (0.99) | *6.14 (0.47)* | 5.84 (0.78) | *0.57* | *0.52* |

0.00001) and for the factor category ($F_{(8-160)}$: 48.67; $p < 0.0001$). No effect was found for the factor 'gender'. Interaction analyses showed a significant interaction between the factor gender and age ($F_{(1-160)}$: 4.02; $p < 0.01$) and between 'age' and 'categories' ($F_{(8-160)}$: 5.81; $p < 0.001$).

Since a significant interaction was found between age and gender and between age and categories, post hoc comparisons between men and women were carried out separately in the young and elderly samples by means of paired T-test for independent sample; data are reported in Table 1.

The comparisons confirmed that (at least within the living categories) significant gender related differences, consistent with the 'experience-dependence hypothesis', can be found in elderly people, because women are more familiar with flowers, whereas men are more acquainted with animals. These asymmetries replicate results obtained in previous studies conducted both in normal subjects and in brain-damaged patients and are in agreement with the 'experience-dependence hypothesis'.

### *3.2. Familiarity ratings obtained in older people considering separately responses given after presentation of verbal and pictorial stimuli*

Since familiarity had been assessed showing both the picture of the target stimulus and the corresponding written name, we computed separately in older people the mean values of familiarity obtained after presentation of stimuli in the verbal and in the pictorial modality.

Data were treated by means of a Three Way ANOVA that considered as independent variable the factors 'gender', 'presentation modality' and 'categories' and as dependent variable the familiarity scores obtained. These data are reported in Table 2.

The analysis showed a significant 'presentation modality' effect ($F_{(1-160)}$: 53.56; $p < 0.0001$) and a significant categories effect ($F_{(8-160)}$: 11.96; $p < 0.0007$). No effect was found for the factor 'gender'. Significant interactions between the factors 'gender' and 'categories' ($F_{(8-160)}$: 1.99; $p < 0.05$) and between 'gender' and 'presentation modality' ($F_{(1-160)}$: 18.24; $p < 0.0002$) were observed.

The study of the single categories showed significant interactions according to the presentation modality for flowers (higher familiarity by name in women) and for the wild animals category (higher familiarity for men by picture and for women by name).

Post hoc comparisons between males and females in the two presentation modalities were carried out by means of Tukey tests. An opposite gender-related trend, with higher familiarity for flowers by word presentation in women and higher familiarity for pets and wild animals by picture presentation in men was again found.

Post hoc comparisons between the two presentation modalities were also carried out separately for men and women by means of Wilcoxon matched pair tests (last two columns of Table 2).

Results confirmed the greater familiarity of women for flowers and of men for animals observed in Table 1, but also showed that an interaction exists between gender and presentation modality in these categories. The greater familiarity of women for flowers is indeed observed after presentation of verbal (but not of pictorial) stimuli, whereas the greater familiarity of men for animals is observed after presentation of pictorial (but not

Table 2
Familiarity of elderly men and women for various categories of knowledge, according to the presentation modality

| Category | Familiarity by picture | | | Familiarity by name | | | p | p | p |
|---|---|---|---|---|---|---|---|---|---|
| | Men | Women | p | Men | Women | p | Interaction | Comparison picture/name women | Comparison picture/name men |
| Fruits | 6.40<br>0.17 | 6.13<br>0.34 | 0.14 | 6.04<br>0.37 | 6.52<br>0.25 | 0.05 | 0.02 | 0.06 | 0.09 |
| Vegetables | 5.84<br>0.35 | 6.01<br>0.29 | 0.43 | 5.83<br>0.44 | 6.02<br>0.29 | 0.46 | 0.73 | 0.99 | 0.99 |
| Flowers | 5.14<br>0.58 | 5.31<br>0.54 | 0.62 | 5.01<br>0.48 | 5.94<br>0.56 | < **0.01** | < **0.001** | < **0.001** | 0.57 |
| Pets | 5.31<br>0.37 | 4.40<br>0.64 | < **0.01** | 5.28<br>0.45 | 5.50<br>0.36 | 0.37 | 0.04 | < **0.006** | 0.99 |
| Wild animals | 3.81<br>0.41 | 2.56<br>0.37 | < **0.001** | 3.63<br>0.34 | 3.93<br>0.30 | 0.12 | < **0.001** | < **0.001** | 0.65 |
| Tools | 6.35<br>0.33 | 5.95<br>0.91 | 0.34 | 6.17<br>0.42 | 6.19<br>0.67 | 0.96 | 0.16 | 0.33 | 0.56 |
| Furniture | 6.18<br>0.48 | 5.98<br>0.63 | 0.59 | 6.02<br>0.61 | 6.49<br>0.66 | 0.27 | 0.04 | 0.03 | 0.65 |
| Clothes | 5.67<br>0.86 | 5.52<br>0.96 | 0.80 | 5.92<br>0.51 | 6.07<br>0.53 | 0.67 | 0.77 | 0.39 | 0.85 |
| Vehicles | 5.07<br>1.08 | 4.17<br>0.93 | 0.20 | 5.19<br>1.05 | 5.23<br>0.93 | 0.95 | 0.03 | < **0.002** | 0.92 |
| All categories | 5.5<br>0.94 | 5.07<br>1.31 | 0.07 | 5.42<br>0.93 | 5.73<br>0.93 | 0.09 | 0.02 | < **0.001** | 0.70 |

In bold are highlighted significant values after Bonferroni correction ($p < 0.01$).

of verbal) stimuli. In females this effect could be due to a general tendency to give higher familiarity ratings after presentation of stimuli in the verbal (mean = 5.73) than in the pictorial (mean = 5.07) modality, whereas in men no difference is observed between familiarity ratings to stimuli presented by name (mean = 5.42) or by picture (mean = 5.50).

## 4. Discussion

Results of this study are more consistent with an 'experience-dependence' interpretation of gender-related asymmetries [16,17,31] than with the assumption that the evolutionary pressures which have prompted the development of different brain networks dedicated to animals and plant life may also have provided each gender with more efficient cognitive representations of their main foraging targets [24,27,29].

As a matter of fact, the main scope of the present investigation consisted in checking if the absence of gender-related familiarity differences for various 'biological' and 'artefact' categories, observed by Gainotti et al. [18] in a study conducted on young undergraduate students could be due to the fact these subjects belonged to a generation in which the traditional social roles have almost completely disappeared. The prediction based on this hypothesis was that, using a very similar procedure, to evaluate the familiarity of older men and women with the same categories of living and the non-living things, we should find in this elderly population significant gender-related familiarity asymmetries, consistent with the 'experience- dependence' hypothesis. Results of our research confirmed these prediction, because data reported in Table 1 showed: (a) that an interaction exists between gender and age and between age and categories with respect to familiarity scores; (b) that in the elderly sample women show a greater familiarity for flowers and men for animals. These findings are consistent with gender-related asymmetries observed in different biological categories by McKenna and Parry [32], Capitani et al. [8], Albanese et al. [1], Laws [29], Marra et al. [31], Barbarotto et al. [3], Cameron et al. [7], Moreno-Martinez et al. [33] and Scotti et al. [35] in normal subjects and by Gainotti [16,17] and by Marra et al. [31] in brain-damaged patients, In our opinion, the greater familiarity of women with flowers is clearly consistent with the 'experience-dependence' hypothesis [13,14] whereas less clearly linked with this assumption is the greater familiarity of men for animals (and particularly for wild animals). An interpretation in favour of this hypothesis had been advanced by Marra et al. [31] in a semantic fluency study in which they had shown that men are more fluent than women with the category 'birds', whereas the opposite asymmetry is observed with the category 'furniture'. To explain the best results obtained by men with 'birds', Marra et al. [31] had noticed that many adult Italian men are hunters and had suggested that their greater proficiency with 'birds' was due to their experience as hunters/fowlers. To verify this hypothesis, Scotti et al. [35] collected familiarity ratings of normal males and females, for a large number of animals, subdivided according to whether they were or not hunted/fished and showed that, even if the overall familiarity was not different between males and females, males were more familiar with hunted animals.

Even though the main goal of our study was achieved, showing that in an old population, we can observe significant gender-related familiarity asymmetries, consistent with the 'experience- dependence' hypothesis, an unexpected finding that deserves a short discussion was that the greater familiarity of women for flowers was significant only after presentation of verbal stimuli, whereas the greater familiarity of men for animals was observed only after presentation of pictorial stimuli.

In females this effect could be due to the general tendency, shown by our women, to give higher familiarity ratings after presentation of stimuli in the verbal than in the pictorial modality, an observation which is consistent with the claim that women are more verbal than men and tend to rely more on verbal than on perceptual data [2,6,22,36]. In men, on the other hand a general trend to give higher familiarity ratings after presentation of stimuli in the pictorial modality was not observed. It is, therefore, possible that their tendency to give higher familiarity rating after pictorial presentation of wild animals was due to the fact that their familiarity with animals was selectively 'visual', as it certainly happens in the context of hunting.

In conclusion, we think that the results of our study give a further support to the experience-dependence' interpretation of gender-related asymmetries, or, in any case, confirm that results obtained in young subjects, which were seemingly at variance with that interpretation, were due to the absence in that experimental sample of the traditional social roles that subsume the different familiarity of men and women with different categories of stimuli.

## Acknowledgments

We had no sources of financial support in the preparation of this manuscript.
